# MiR-596 inhibits osteoblastic differentiation and cell proliferation by targeting Smad3 in steroid-induced osteonecrosis of femoral head

**DOI:** 10.1186/s13018-020-01688-5

**Published:** 2020-05-14

**Authors:** Ligong Fu, Huawei Liu, Weijun Lei

**Affiliations:** 1 Department of Orthopaedic Surgery, Beijing Tsinghua Changgung Hospital, School of Clinical Medicine, Tsinghua University, Beijing, 102218 China; 2 Department of Orthopaedic Surgery, Hongze Huaian District People’s Hospital, No. 102 Dongfeng Road, Hongze District, Huai’an City, 223100 Jiangsu Province China

**Keywords:** Steroid-induced osteonecrosis of femoral head, miR-596, Smad3, Osteoblastic differentiation, Proliferation

## Abstract

**Background:**

It is reported that miR-596 has a potential diagnostic value for non-traumatic osteonecrosis of the femoral head (NOFH), but its underlying mechanisms in NOFH is unclear.

**Methods:**

The expression of miR-596 and Smad3 was detected by western blot and quantitative real-time PCR. The relationship between the two molecules was explored using Dual-Luciferase Reporter Assay. Glucocorticoid (GC)—dexamethasone, was used to induce bone marrow mesenchymal stem cell (BMSC) osteogenic differentiation, and the effects of miR-596 on BMSC osteogenic differentiation and proliferation were determined.

**Results:**

MiR-596 expression was upregulated, while Smad3 expression was inhibited in the bone marrow samples of patients with steroid-induced osteonecrosis of femoral head (SANFH). Overexpression of miR-596 inhibited the proliferation and osteogenic differentiation of BMSCs induced by GC. Meanwhile, the opposite results were observed in the miR-596 inhibitor group. In addition, Smad3 was a target gene of miR-596, and negatively regulated by miR-596. The promotion effect of the miR-596 inhibitor on BMSC proliferation and osteogenic differentiation was reversed by si-Smad3.

**Conclusion:**

MiR-596 can suppress GC-BMSC osteoblastic differentiation and proliferation by regulating Smad3 expression.

## Background

Non-traumatic osteonecrosis of the femoral head (NOFH) is a progressive pathological process that results in osteonecrosis and collapse due to the blocked blood supply of the femoral head, the interruption of local circulation, and the impairment of the blood supply to the subchondral bone, ultimately leading to impaired hip function and permanent disability [1, 2]. Patients always miss the best treatment opportunity because the early symptoms of NOFH are not obvious [3]. Some studies have shown that glucocorticoid (GC), which is widely used in various diseases such as severe acute respiratory syndrome, systemic lupus erythematosus, rheumatoid arthritis, and leukemia, is an important predisposing factor for steroid-induced osteonecrosis of femoral head (SANFH) [4]. In addition, it has been confirmed that high doses of glucocorticoids are associated with the occurrence of femoral head necrosis [5]. However, there are no effective preventive measures for SANFH.

Bone marrow mesenchymal stem cells (BMSCs) are a group of stem cells with self-renewal and differentiation capabilities, which can differentiate into various cells, including adipocytes, chondrocytes, and osteoblasts [6]. Previous studies demonstrated that the change in osteogenic differentiation of BMSCs is related to SANFH [7, 8]. It has been proved that the osteoblastic differentiation of BMSCs in SANFH is alleviated, but the fat differentiation is promoted [9]. In addition, the proliferation ability of BMSCs is reduced in patients with SANFH [10]. Therefore, the search for molecules that can regulate the proliferation and osteoblastic differentiation of BMSCs is critical for identifying new targets for the prevention of SANFH.

MicroRNAs (miRNAs), as a kind of endogenous non-coding RNA, can participate in the regulation of physiological processes in various cells, including BMSCs [11]. For example, it was reported that overexpression of miR-149-3p could promote BMSC osteogenic differentiation [12]. In addition, some miRNAs have been found to be involved in the regulation of SANFH, such as miR-708 [13], miR-27a [14], and miR-195-5p [15]. MiR-596 is a kind of miRNAs, which was indispensable for the differentiation, proliferation, and migration of cancer cells [16, 17]. It was reported that plasma miR-596 level was significantly increased in patients with NOFH, which had potential diagnostic value for NOFH [18]. However, the underlying mechanisms of miR-596 in SANFH are unclear.

SMAD protein family belongs to the intracellular signaling protein, which was first identified in invertebrates by gene screening [19]. Studies have shown that the SMAD protein family plays a key role in bone formation [20, 21]. Smad3 is a member of the SMAD protein family, which has been proved to be a critical factor in the normal maintenance of the bone [22]. For example, Borton et al. revealed that Smad3 knockout mice had osteopenia after weaning [23]. Additionally, Smad3 can also participate in the proliferation and osteogenic differentiation of BMSCs [24]. Previous studies have suggested that lncRNA H19 can promote BMSC osteogenic differentiation, which is related to Smad3 [25]. The role of transforming growth factor-beta 1 in BMSC proliferation is also associated with Smad3 [26].

Generally, miRNAs exert their role through binding to the 3′-untranslated region (3′-UTR) of target genes to regulate the expression of target genes [27]. It has been proved that miR-181d and miR-708 can regulate the osteogenic differentiation of BMSCs by targeting Smad3 [5, 13]. Thus, we speculate the role of miR-596 in SANFH may be related to Smad3.

Herein, we investigated the relationship between miR-596 and Smad3, as well as the underlying mechanisms of miR-596 in SANFH, aiming to discover a novel target for the prevention and treatment of SANFH.

## Methods

### Samples

Twenty bone marrow samples from patients with SANFH (SANFH group) and 20 bone marrow samples from patients with femoral neck fracture who underwent total hip replacement (control group) were collected from the Hongze Huaian District People’s Hospital. According to the Ficat and Arlet staging system, all patients with SANFH were classified as phases II, III, or IV. All patients are between the ages of 25 and 50. Patients with metabolic bone disease, pathological fractures, genetic disease, and a history of infectious disease were excluded. Informed consent was taken from all patients. All experimental protocols conformed to the World Medical Association Declaration of Helsinki and were approved by the Ethics Committee at Hongze Huaian District People’s Hospital (Approval no.2019005).

### Cell culture and treatment

BMSCs (ATCC, Manassas, VA, USA) were maintained in a complete medium containing with 100 U/ml penicillin, 10% fetal bovine serum, and 100 μg/ml streptomycin (all from Gibco; Waltham, USA) and cultured at 37 °C and 5% CO_2_. Different concentrations (10^-8^ M, 10^-7^ M, and 10^-6^ M) of GC—dexamethasone (Dex), was added into the medium, and miR-596 expression was measured by quantitative real-time PCR (qRT-PCR). The cell morphology of BMSCs n the first and fourteenth day after GC induction was observed under an inverted microscope. MiR-596 mimics, miR-596 inhibitor, si-Smad3, and their negative control (NC) were procured from Shanghai GenePharma, Co., Ltd. (China). Following the manufacturer’s protocol, cell transfections were carried out using Lipofectamine 2000 (Invitrogen, Carlsbad, USA).

### qRT-PCR

Trizol reagent (Invitrogen) was used to extract total RNAs from cells. Transcriptase Kit (Takara, Otsu, Japan) was then used for reversing cDNA. qRT-PCR was carried out by SYBR Green PCR Master Mix (Takara) with the ABI StepOnePlus Real-Time PCR system (Applied Biosystems, Foster City, CA, USA). The molecule expressions were calculated using 2^-ΔΔCt^ method. Each sample was performed in triple. The sequences of primers were listed in Table 1.

**Table 1:** List of primer sequences were used in the study

Name	Primer	Sequence (5′-3′)
miR-596	Forward	AAGCCTGCCCGGCTCCT
	Reverse	GCTGTCAACGATACGCTACGT
Smad3	Forward	CCTCTCCAGCAATAATCCGAA
	Reverse	TGCCCAATTTTCTTTACCAGT
ALP	Forward	TGACCTTCTCTCCTCCATCC
	Reverse	CTTCCTGGGAGTCTCATCCT
OPN	Forward	CCAAGCGTGGAAACACACAGCC
	Reverse	GGCTTTGGAACTCGCCTGACTG
Runx-2	Forward	ACTTCCTGTGCTCGGTGCT
	Reverse	GACGGTTATGGTCAAGGTGAA
Osterix	Forward	GGCACAAAGAAGCCGTACTC
	Reverse	GCCTTGTACCAGGAGCCATA
U6	Forward	CGCTTCGGCAGCACATATAC
	Reverse	TTCACGAATTTGCGTGTCAT
GAPDH	Forward	TCGACAGTCAGCCGCATCTTCTTT
	Reverse	GCCCAATACGACCAAATCCGTTGA

### Western blotting

RIPA lysis buffer (Beyotime, Beijing, China) was used to extract total protein from cells, and a BCA protein assay kit (Beyotime) was used to measure the protein concentrations following the manufacturer’s protocols. The protein samples were separated and transferred using 12% SDS-PAGE and PVDF membranes. The membranes were blocked in skimmed milk (5%) at room temperature for 2 h. After that, the membranes were cultured with primary antibodies at 4 °C overnight. Primary antibodies includes anti-Smad3 antibody (1:1000, Abcam), anti-ALP antibody (1:1000, Abcam), anti-Osteopontin (OPN) antibody (1:1000, Abcam), anti-Runx-2 antibody (1:1000, Abcam), anti-Osterix antibody (1:1000, Abcam), and anti-GAPDH antibodies (1:2000, Abcam). In the next day, the membranes were incubated with a horseradish peroxidase-conjugate secondary antibody (1:2000, Santa Cruz) for 1 h at room temperature. The enhanced chemiluminescence detection system (ECL, Roche Molecular Biochemicals) was used to measure the blots.

### Methyl thiazolyl tetrazolium (MTT) assay

Cell proliferation ability was detected by MTT assay. Briefly, cells were seeded into 96-well plates (1 × 10^3^ cells/ml) in each well and cultured for 3 days. MTT solution (15 μl, 5 mg/ml) was then added into each well. Four hours later, the supernatants were removed. To dissolve the resultant formazan crystals, DMSO (150 μl/per well) was added to each well. A spectrophotometer was used to measure the absorption at 570 nm.

### Flow cytometry

After digesting, BMSCs were resuspended in PBS (10^6^ cells/ml). Then, cell suspension (500 μl) was incubated with anti-CD44 antibody (1:40, Abcam) or anti-CD45 antibody (1:20, Abcam) in the dark for 30 min at room temperature. Subsequently, cells were resuspended in PBS (500 μl). The cellular phenotype of BMSCs was detected using flow cytometry.

### Osteoblastic differentiation induction

To induce osteoblastic differentiation, the culture medium of BMSCs was changed to osteoblastic differentiation medium which included the basal medium, 0.2 mM ascorbate, 0.1 μM Dex, and 10 mM β-glycerophosphate.

### Alkaline phosphatase (ALP) staining

Cells were washed with PBS three times and then fixed with 4% paraformaldehyde. After 30 min, the ALP staining solution was added and cultured for 1 h at 37 °C. Subsequently, cells were rinsed for 2 min and observed under an inverted optical microscope.

### Alizarin red staining

The mineralized nodule formation was detected using Alizarin red staining. Briefly, after fixing with 4% paraformaldehyde for 10 min, cells were stained with Alizarin red solution (1%) at room temperature for 30 min. Then, cells were washed with PBS, dried, and observed under a microscope.

### Dual-luciferase reporter assay

Smad3 3′UTR sequence containing wild type (WT) or mutant (MUT) miR-596 putative binding region was amplified by RiboBio (Guangzhou, China) and inserted into pGL3-GP73-3′UTR plasmid (Invitrogen). Then, the plasmids and miR-596 mimics (or miR-NC) were co-transfected into cells using Lipofectamine 2000 (Invitrogen). The Dual-Luciferase Reporter Assay System (Promega, USA) was performed to measure the luciferase activity after 48 h of transfection.

## Statistical analysis

Statistical analysis was calculated by SPSS 19.0 (SPSS, Chicago, IL, USA). Mean ± standard deviation was used to present the measurement data. The difference between the two groups and the difference in multiple groups were compared using Student’s *t* test and ANOVA, respectively. The correlation between miR-596 and Smad3 was analyzed using Pearson correlation analysis. All assays were performed at least in triplicate. *P* < 0.05 was considered statistically significant.

## Results

### MiR-596 expression was upregulated while Smad3 expression was inhibited in SANFH

**Fig. 1:**
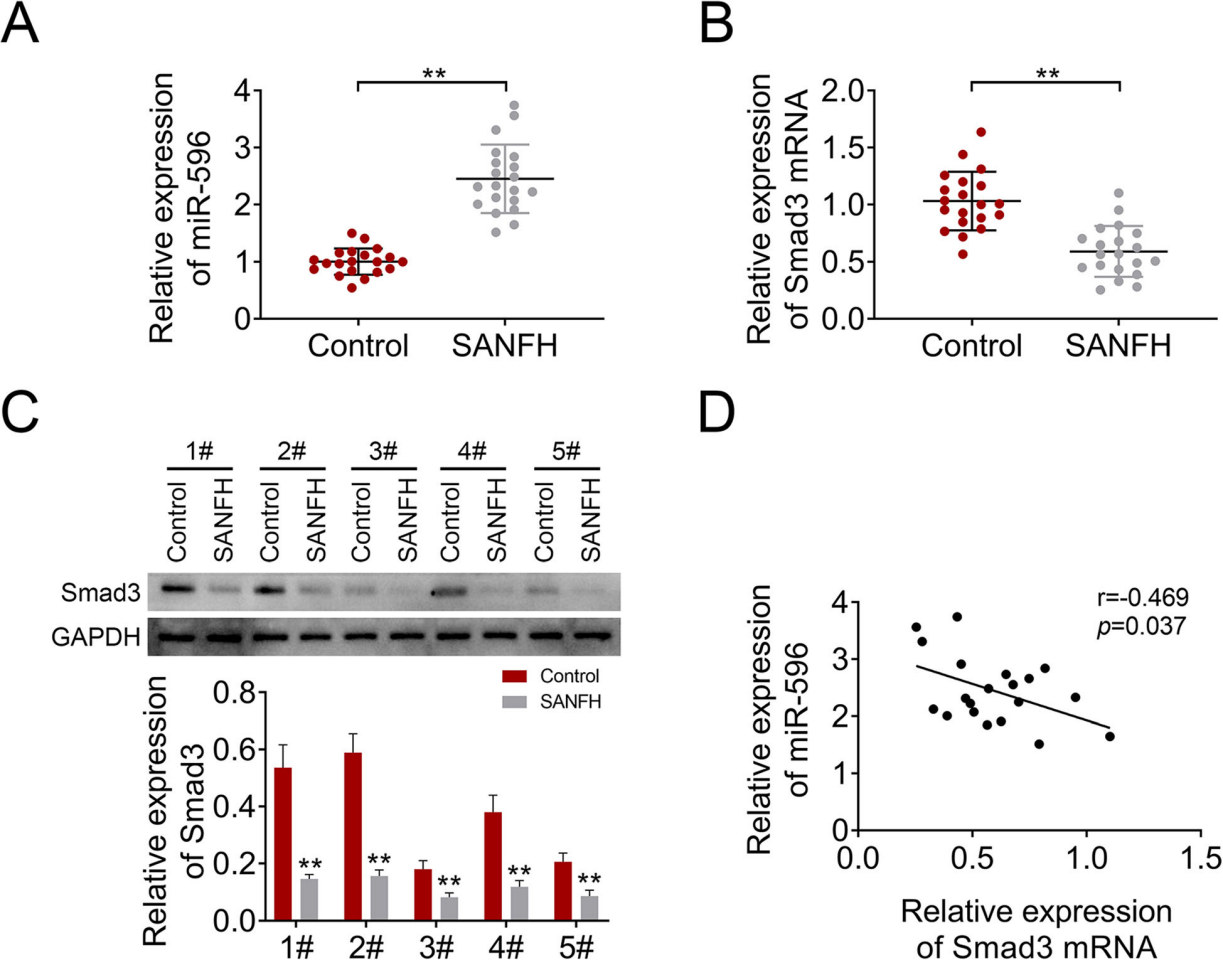
The expression of miR-596 and Smad3 in bone marrow samples of patients with SANFH. **a** MiR-596 expression was examined by qRT-PCR. **b** The mRNA level of Smad3 was determined using qRT-PCR. **c** The protein level of Smad3 was determined using western blotting. **d** The correlation analysis of miR-596 and Smad3. **P < 0.01 vs control

We first measured the expression of miR-596 and Smad3 in SANFH. As shown in Fig. 1a, miR-596 expression in samples of SANFH was higher compared to the control group. Conversely, the mRNA and protein levels of Smad3 in the SANFH group were lower compared with the control group (Fig. 1b, c). The correlation analysis showed that the expression of mir-596 and Smad3 was negatively correlated in SANFH (Fig. 1d). These results indicated that miR-596 and Smad3 might be related with SANFH.

### MiR-596 expression was increased in GC-BMSCs

**Fig. 2:**
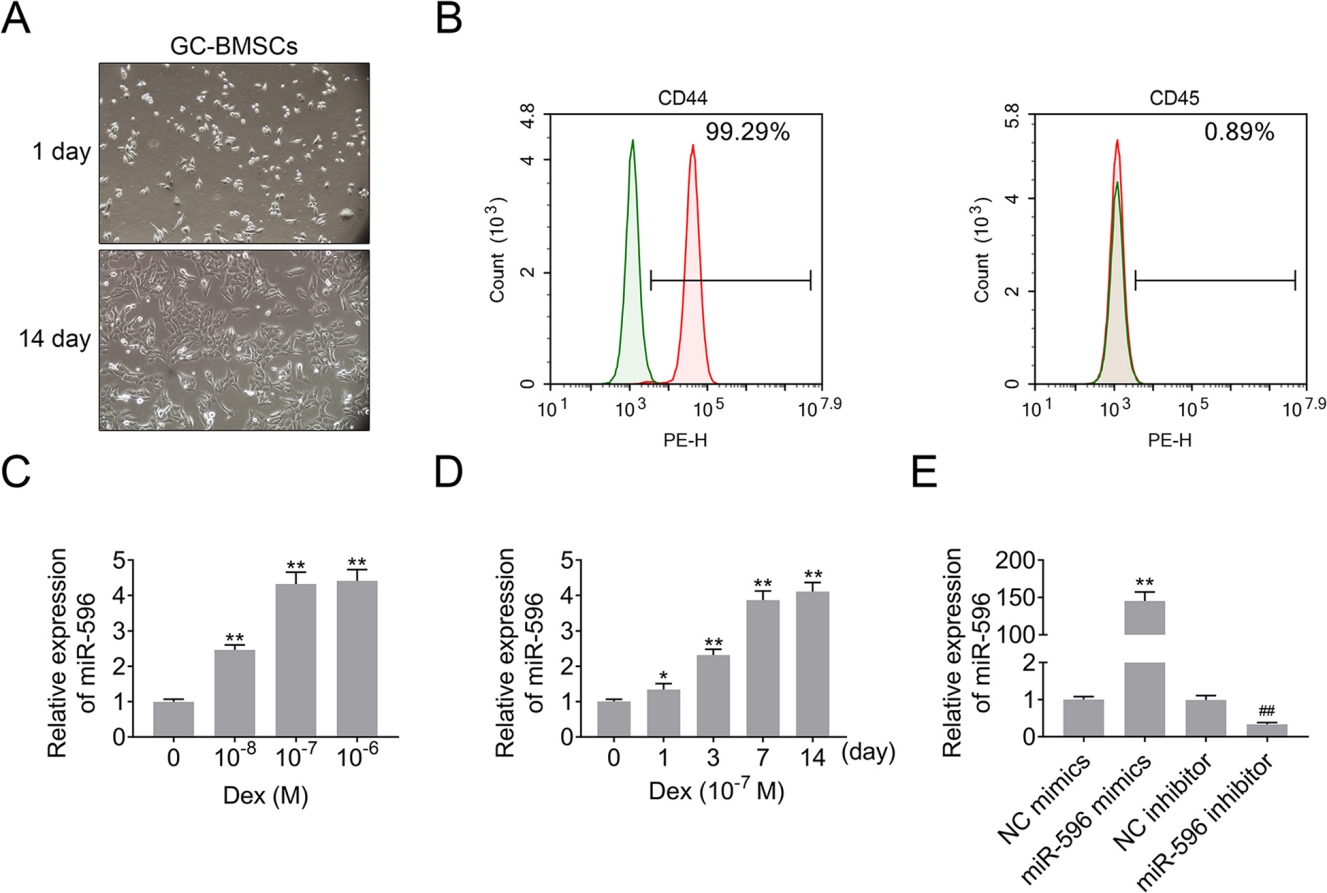
MiR-596 expression in GC-BMSCs. **a** The shape of GC-BMSCs was observed under an inverted microscope. **b** The BMSC markers (CD44 and CD45) were examined using flow cytometry. **c** MiR-596 expression in BMSCs induced by different concentrations of Dex (gradient concentration: 10^-8^ M, 10^-7^ M, and 10^-6^ M). **d** The expression level of miR-596 in BMSCs induced by Dex (10^-7^ M) in different induction time. **e** MiR-596 expression in BMSCs transfected with miR-596 mimics or miR-596 inhibitor. **P < 0.01 vs 0 M, 0 day, or NC mimics. ^##^*P* < 0.01 vs NC inhibitor

Herein, we tested the expression of miR-596 in GC-BMSCs. Firstly, we observed the shape of BMSCs induced by GC. The results of an inverted microscope showed that GC-BMSCs grew in a short fusiform or star-shaped dispersion adherent after 1 day of primary culture (Fig. 2a). After 14 days, GC-BMSCs were arranged in a sequence along the long axis of the cell body and presented a vortex shape (Fig. 2a). Subsequently, the BMSC markers (CD44 and CD45) were examined using the flow cytometry to test the purity of BMSCs. As shown in Fig. 2b, CD44 (99.29%) was positively expressed, and CD45 (0.89%) was negatively expressed in BMSCs. Then, qRT-PCR detected miR-596 expression in BMSCs induced by different concentrations of Dex (gradient concentration: 10^-8^ M, 10^-7^ M, and 10^-6^ M), and the results suggested that miR-596 expression was enhanced with the increase of Dex concentration (Fig. 2c). Additionally, the expression level of miR-596 in BMSCs induced by 10^-7^ M Dex was similar to that in BMSCs induced by 10^-6^ M Dex (Fig. 2c); thus, 10^-7^ M Dex was selected for the following experiments. As shown in Fig. 2d, the expression level of miR-596 in BMSCs was increased with the time of Dex (10^-7^ M) induction. Moreover, miR-596 inhibitor downregulated miR-596 expression in BMSCs, while miR-596 mimics upregulated miR-596 expression (Fig. 2e). Taken together, the results revealed that GC could increase miR-596 expression in BMSCs.

### MiR-596 inhibited GC-BMSC proliferation and osteogenic differentiation

**Fig. 3:**
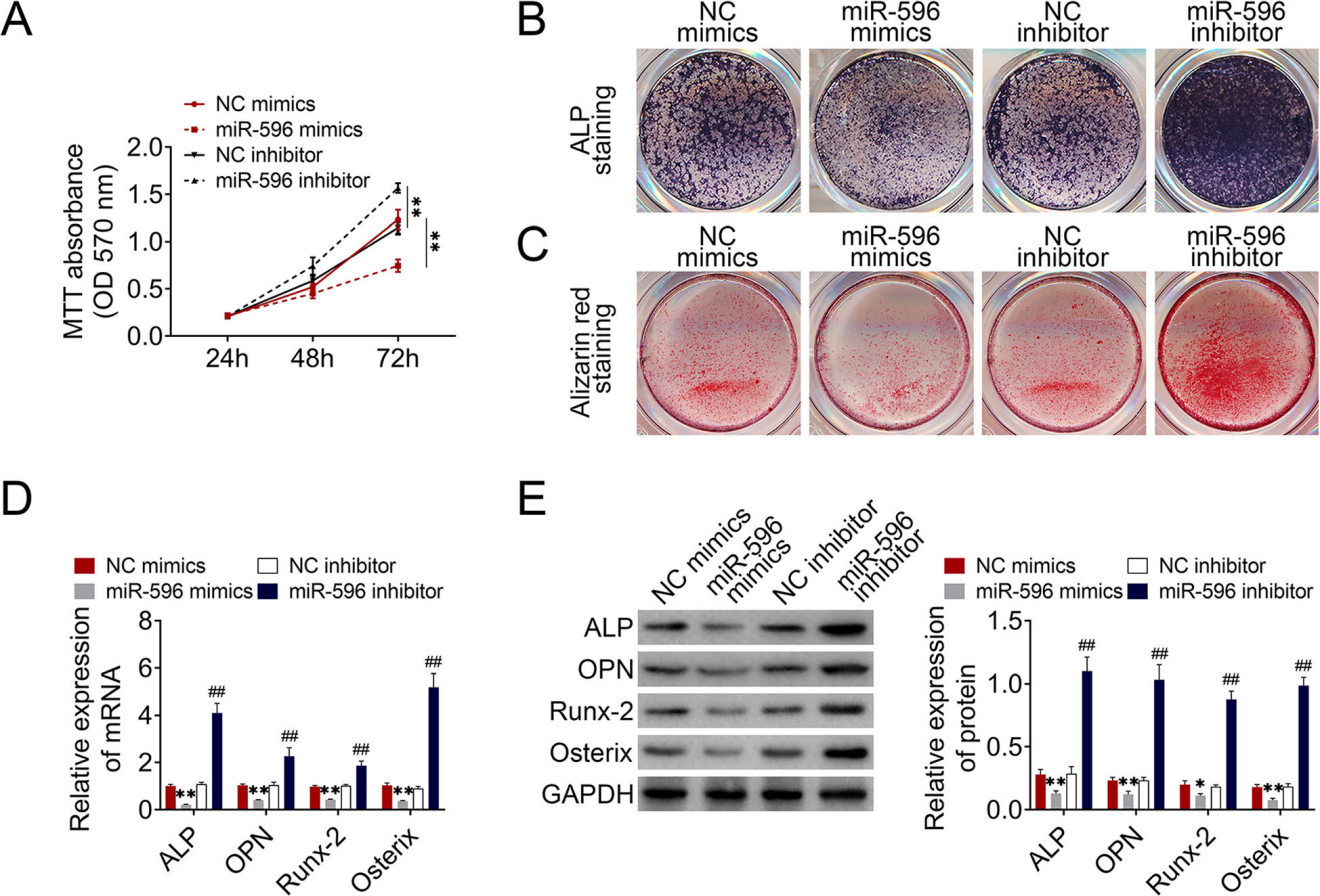
MiR-596 regulated GC-BMSC proliferation and osteogenic differentiation. GC-BMSCs were transfected with miR-596 mimics or miR-596 inhibitor. **a** The proliferation ability of GC-BMSCs was measured using MTT assay. **b** ALP staining. **c** Alizarin red staining. **d** The mRNA expression of ALP, OPN, Runx-2, and Osterix was determined using qRT-PCR**. e** The protein level of ALP, OPN, Runx-2, and Osterix was determined using western blotting. **P < 0.01 vs NC mimics. ^##^*P* < 0.01 vs NC inhibitor

To explore the function of miR-596 on BMSCs, we transfected miR-596 mimics and miR-596 inhibitor into GC-BMSCs. MTT results suggested that the proliferation ability of GC-BMSCs with upregulated miR-596 was subdued, while the ability was enhanced in the miR-596 inhibitor group (Fig. 3a). ALP staining and alizarin red staining results revealed that GC-BMSCs in the miR-596 mimic group showed lighter ALP staining and less alizarin red-stained mineralized nodules than the NC mimic group, whereas GC-BMSCs in miR-596 inhibitor group showed darker ALP staining and more alizarin red-stained mineralized nodules than NC inhibitor group (Fig. 3b, c). In addition, qRT-PCR and western blotting showed a low expression of ALP, OPN, Runx-2, and Osterix in the miR-596 mimic group, but a high expression of these molecules in the miR-596 inhibitor group (Fig. 3d, e). The above findings suggested that miR-596 negatively regulated GC-BMSC proliferation and osteogenic differentiation.

### Smad3 was a target gene of miR-596

**Fig. 4:**
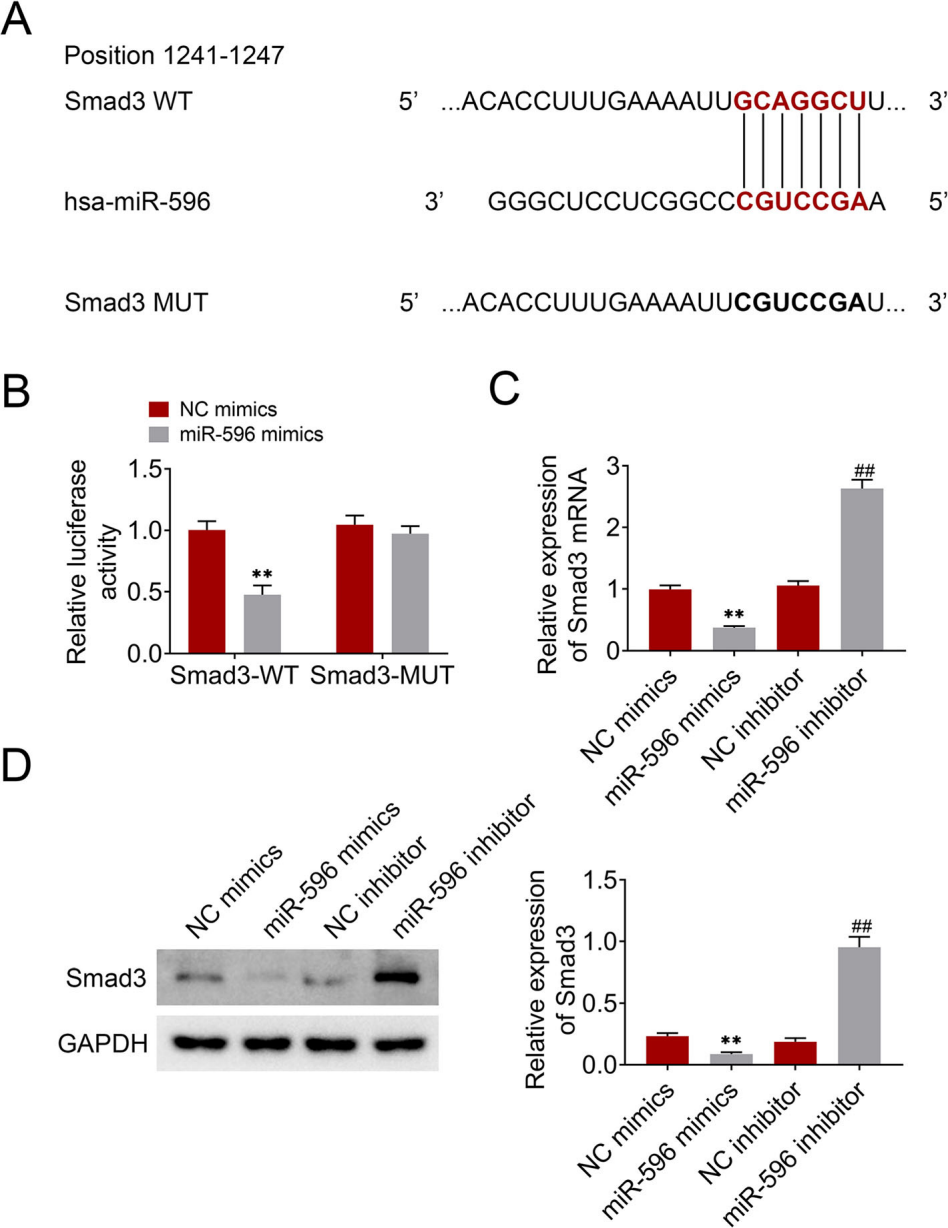
Smad3 was a target gene of miR-596. **a** The binding sites between miR-596 and Smad3 3′-UTR were predicted through Targetscan. **b** Dual-Luciferase Reporter Assay was used to explore the relationship between miR-596 and Smad3. **c** The influence of miR-596 on the Smad3 mRNA level. **d** The influence of miR-596 on the Smad3 protein level. **P < 0.01 vs NC mimics. ^##^*P* < 0.01 vs NC inhibitor

To investigate the miR-596 mechanism in BMSCs, TargetScan (http://www.targetscan.org/vert_72/) was used to predict the target gene of miR-596 and found Smad3 (Fig. 4a). Dual-Luciferase Reporter Assay showed that the co-transfection of miR-596 mimics inhibited the luciferase activity of Smad3-WT (Fig. 4b). Moreover, the effects of miR-596 on Smad3 expression in BMSCs were determined. The results demonstrated that Smad3 expression was downregulated by overexpression of miR-596 and was upregulated by knockdown of miR-596 (Fig. 4c, d). These data revealed that Smad3 was negatively regulated by miR-596.

### MiR-596 participated in GC-BMSC proliferation and osteogenic differentiation through regulating Smad3

**Fig. 5:**
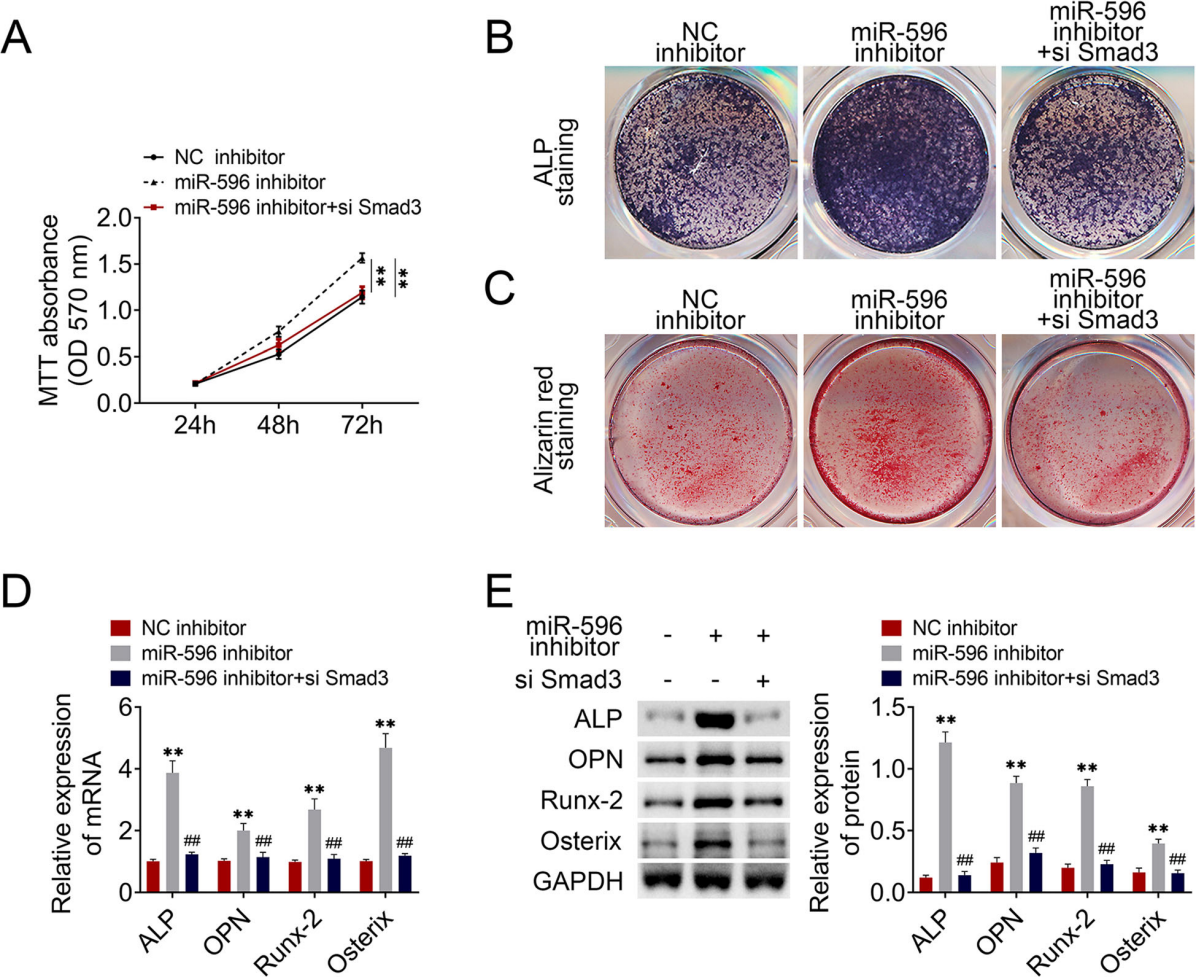
MiR-596 regulated GC-BMSC proliferation and osteogenic differentiation through regulating Smad3. **a** MTT assay. **b** ALP staining. **c** Alizarin red staining. **d** The mRNA expression of ALP, OPN, Runx-2, and Osterix was determined using qRT-PCR**. e** The protein level of ALP, OPN, Runx-2, and Osterix was determined using western blotting. **P < 0.01 vs NC inhibitor. ^##^*P* < 0.01 vs miR-596 inhibitor

To reveal the function of miR-596/Smad3 on BMSCs, miR-596 inhibitor and si-Smad3 were transfected into GC-BMSCs. As shown in Fig. 5a, the promotion effect of the miR-596 inhibitor on GC-BMSC proliferation was reversed by silencing Smad3. Furthermore, the ALP staining of GC-BMSCs became darker and alizarin red staining was enhanced in the miR-596 inhibitor group, while si-Smad3 changed the trend (Fig. 5b, c). The mRNA and protein expression of ALP, OPN, Runx-2, and Osterix was increased by repressing miR-596 and was reduced via si-Smad3 (Fig. 5d, e). Above data revealed that the function of miR-596 in GC-BMSC proliferation and osteogenic differentiation was related with Smad3.

## Discussion

Osteonecrosis of the femoral head (ONFH), also known as avascular necrosis of the femoral head, is a chronic and highly disabling disease that destroys the blood circulation of the femoral head due to various reasons, causes the death of bone cells and bone marrow components, and ultimately leads to osteonecrosis of the femoral head cartilage, collapse of the femoral head, and dysfunction of the hip joint [28, 29]. ONFH is classified as traumatic and non-traumatic. Long-term use of high-dose glucocorticoids is the most common cause of NOFH, and it occurs mostly in young adults [30]. At present, the clinical treatment of NOFH includes conservative treatment (biophysical stimulation such as pulsed electromagnetic field) and surgical methods (such as core decompression), but the effect is not significant [31]. In addition, most patients undergo one or more artificial joint revision operations, which imposes serious economic and mental burdens on society and patients’ families [32]. Thus, it is urgent to find new strategies for the prevention and treatment of NOFH.

One of the main causes of SANFH is the stagnation of osteoblast differentiation and osteopenia [33], so regulating this process has profound significance for SANFH treatment. BMSCs are important stromal cells in osteogenesis and metabolism [34]. It has revealed that miR-149-3p and Runx1 can reduce the adipogenic differentiation of BMSCs and enhance the osteogenic differentiation [12, 35]. In SANFH, the researchers found that BMSCs had specific differentiation to adipocytes, while the differentiation to osteoblasts was basically stagnant [14]. In addition, Huang et al. suggested that Icariin could improve the balance between osteogenic differentiation and adipogenic differentiation of BMSCs and then reduce the bone loss of SANFH rats [36]. These findings indicate that BMSCs are the key cytological basis for the pathogenetic mechanism of SANFH [6]. Therefore, exploring the molecules involved in the process of BMSC differentiation is expected to provide new ideas for the diagnosis and treatment of SANFH.

It has been confirmed that the abnormal expression of miRNAs is closely related to stem cell differentiation [37]. For instance, miR-34a, miR-450a-5p, and miR-28-5p have been revealed to be involved in regulating the osteoblastic differentiation of mesenchymal stem cells [38, 39]. In BMSCs, the adipogenic and osteogenic differentiation can also be regulated by miR-383 [40], miR-20a-5p [41], and miR-20a-5p [41]. Moreover, some miRNAs are reported to be involved in the development of SANFH. For example, miR-27 could promote BMSC proliferation and osteogenic differentiation in SANFH [42], and miR-144-3p could inhibit the proliferation and osteogenic differentiation of BMSCs in SANFH [43]. These suggest that miRNAs can participate in the process of SANFH by regulating BMSCs.

As a miRNA located on human chromosome 8p23.3, miR-596 acts as an antioncogene in a variety of tumors [44]. Endo et al. demonstrated that miR-596 inhibited the growth of oral cancer through targeting LGALS3BP [45]. Wei et al. suggested that miR-596 exerted the role in glioma invasion via regulating CREPT [46]. In patients with SANFH, miR microarray analysis showed that miR-596 level in plasma was significantly elevated [18]. However, the function of miR-596 in SANFH remains to be elucidated. In the present study, we found that miR-596 was strongly expressed in bone marrow samples of patients with SANFH. Similarly, GC treatment could increase miR-596 expression in BMSCs. Moreover, GC-BMSC proliferation and osteogenic differentiation could be negatively regulated by miR-596. These findings indicated that miR-596 had an important role in SANFH development. To clarify this effect, we further studied the mechanism of miR-596 in SANFH.

Based on the bioinformatics analysis of the miRNA target database, we found that Smad3 was a target gene of miR-596. As a member of the SMAD family, Smad3 has been reported to participate in regulating multiple cellular functions, including BMSC osteogenic differentiation. For instance, TGFβ signaling regulated the osteoblastic differentiation of BMSCs through Smad3 [47], and overexpression of lncRNA H19 promoted BMSC osteogenic differentiation via TGF-β1/Smad3/HDAC signaling pathway [25]. In addition, Smad3 can also be involved in the regulation of SANFH. Hao and his colleagues revealed that miR-708 could inhibit osteogenic differentiation and promote SANFH via downregulating Smad3 [13]. In the current study, we discovered that miR-596 could negatively regulate the mRNA and protein level of Smad3, and knockdown of miR-596 showed enhancement of osteogenic differentiation and proliferation capability by increasing Smad3 in GC-BMSCs, suggesting that miR-596 might be involved in the regulation of osteogenic differentiation in SANFH. However, due to time and funding problems, the sample size of this study is small, and we will collect as many samples as possible to verify later.

## Conclusions

Collectively, we are the first to demonstrate that knockdown of miR-596 could promote the proliferation and osteogenic differentiation of GC-BMSCs, and the function was related to the upregulation of Smad3. Modulation of miR-596 might be an innovative method for the prevention and treatment of SANFH.

## Data Availability

All data generated or analyzed during this study are included in this published article.

## Abbreviation

ALP: Alkaline phosphatase
BMSCs: Bone marrow mesenchymal stem cells
Dex: Dexamethasone
GC: Glucocorticoid
NOFH: Non-traumatic osteonecrosis of the femoral head
ONFH: Osteonecrosis of the femoral head
OPN: Osteopontin
RUNX2: Runt-related transcription factor 2
SANFH: Steroid-induced osteonecrosis of femoral head
